# Age Stereotypes and Stereotypes of Persons Living With Memory Loss With Health in Parent-Adult Child Dyads

**DOI:** 10.1093/geroni/igaf122.3530

**Published:** 2025-12-31

**Authors:** Yunke Gu, Joan Monin, Becca Levy, Marianne LaFrance, Heidi Gil

**Affiliations:** Yale School of Public Health, New Haven, Connecticut, United States; Yale, Yale, Connecticut, United States; Yale School of Public Health, New Haven, Connecticut, United States; Yale, New Haven, Connecticut, United States; LiveWell, Plantsville, Connecticut, United States

## Abstract

Negative stereotypes of aging have been associated with poor overall health for the people that hold them. However, research examining stereotypes of persons living with memory loss and how they relate to health remains limited. Little is known about interpersonal influence of these stereotypes within intergenerational caregiving dyads. This study examines the dyadic associations between self-perceptions of aging and persons living with memory loss, and health outcomes in parent-adult child caregiving dyads. This study is a secondary analysis of 126 dyads from the Families Coping Together with Alzheimer’s Disease study. Measures included self-reported depressive symptoms and physical and mental health. Positivity scores of age stereotypes and stereotype of persons living with memory loss were derived from participant self-reported words coded on a 5-point scale by independent raters. Actor-Partner Interdependence Models were used to examine intrapersonal and interpersonal associations between the dyads. Our dyadic analyses indicate that positive age stereotypes were significantly associated with better mental health and fewer depressive symptoms for both dyad members (actor effects). Positive stereotypes of people living with memory loss were associated with greater physical health in both dyad members (actor effects). No partner effect emerged. Parent and child stereotypes were not significantly associated. The study underscores the role of self-perceptions of aging and memory loss in shaping individual health outcomes within the context of caregiving dyads. While stereotypes may be more impactful for health intrapersonally than interpersonally, fostering positive perceptions on aging and memory loss may support well-being in caregiving dyads and broader society.

